# Hypoxia‐inducible factor 1‐alpha acts as a bridge factor for crosstalk between ERK1/2 and caspases in hypoxia‐induced apoptosis of cementoblasts

**DOI:** 10.1111/jcmm.16920

**Published:** 2021-09-14

**Authors:** Jiawen Yong, Julia von Bremen, Sabine Groeger, Gisela Ruiz‐Heiland, Sabine Ruf

**Affiliations:** ^1^ Department of Orthodontics Faculty of Medicine Justus Liebig University Giessen Giessen Germany; ^2^ Department of Periodontics Faculty of Medicine Justus Liebig University Giessen Giessen Germany

**Keywords:** adipokines, apoptosis, cementoblasts, cobalt (II) chloride, hypoxia

## Abstract

Hypoxia‐induced apoptosis of cementoblasts (OCCM‐30) may be harmful to orthodontic treatment. Hypoxia‐inducible factor 1‐alpha (HIF‐1α) mediates the biological effects during hypoxia. Little is known about the survival mechanism capable to counteract cementoblast apoptosis. We aimed to investigate the potential roles of HIF‐1α, as well as the protein‐protein interactions with ERK1/2, using an in‐vitro model of chemical‐mimicked hypoxia and adipokines. Here, OCCM‐30 were co‐stimulated with resistin, visfatin or ghrelin under CoCl_2_‐mimicked hypoxia. In‐vitro investigations revealed that CoCl_2_‐induced hypoxia triggered activation of caspases, resulting in apoptosis dysfunction in cementoblasts. Resistin, visfatin and ghrelin promoted the phosphorylated ERK1/2 expression in OCCM‐30 cells. Furthermore, these adipokines inhibited hypoxia‐induced apoptosis at different degrees. These effects were reversed by pre‐treatment with ERK inhibitor (FR180204). In cells treated with FR180204, HIF‐1α expression was inhibited despite the presence of three adipokines. Using dominant‐negative mutants of HIF‐1α, we found that siHIF‐1α negatively regulated the *caspase*‐*8*, *caspase*‐*9* and *caspase*‐*3* gene expression. We concluded that HIF‐1α acts as a bridge factor in lengthy hypoxia‐induced apoptosis in an ERK1/2‐dependent pathway. Gene expressions of the *caspases*‐*3*, *caspase*‐*8* and *caspase*‐*9* were shown to be differentially regulated by adipokines (resistin, visfatin and ghrelin). Our study, therefore, provides evidence for the role of ERK1/2 and HIF‐1α in the apoptotic response of OCCM‐30 cells exposed to CoCl_2_‐mimicked hypoxia, providing potential new possibilities for molecular intervention in obese patients undergoing orthodontic treatment.

## 1 INTRODUCTION

During orthodontic tooth movement (OTM), compressive forces create a specific microenvironment^1^ in which cementoblasts are exposed to reduced levels of oxygen resulting in locally decreased cellular oxygen tension, so‐called hypoxia.^2^ The effects of hypoxia on periodontal ligament cell have previously been investigated using hypoxia chambers.^3^ However, it is difficult to maintain a local steady oxygen tension through this method. Alternatively, several metals have been proven to be hypoxia mimicking agents: cobalt (II) chloride^4^ and nickel chloride.^5^ The chemically induced hypoxia alters cell behaviour^6^ and induces apoptosis after long exposure periods.^7^, ^8^ Correspondingly, Wu et al. (2013) reported that CoCl_2_‐mimicked hypoxia can induce apoptosis in cementoblasts.^9^

CoCl_2_ imitates hypoxia in vitro by preventing the hypoxia‐inducible factor‐alpha (HIF‐1α) from being destroyed by oxygen.^10^ Kanaya et al. (2003) reported that CoCl_2_ treatment induces HIF‐1α expression stability.^11^ CoCl_2_ was proven to participate in multiple cellular responses to produce oxidative stress, induce cell damage, reduce cell mitochondrial membrane potential, activate the caspase family and ultimately induce apoptosis.^12^ Song et al. (2012) showed that CoCl_2_ could induce cytotoxicity through mitochondria‐apoptotic and autophagic pathways involved in HIF‐1α in human periodontal ligament cells in‐vitro.^7^

Obese patients have an impaired cementum metabolism, due in part to their altered levels of circulating adipokines.^13^, ^14^ Adipokines, produced mainly by adipocytes, which influence apoptosis and inflammatory responses of cementoblasts during OTM in obese individuals.^15^ Thus, it is of vital importance to investigate the influence of adipokines on the apoptosis of cementoblasts under the experimental hypoxic setup.

CoCl_2_‐mimicked hypoxia has been reported to correlate with apoptotic and pro‐apoptotic factors,^16^ effects that are dependent on target genes regulated by HIF‐1α.^17^ Based on this biological characteristic,^18^ the relation between hypoxia and apoptosis is now the subject of considerable research, but the effects of adipokines on the hypoxia‐induced apoptosis of cementoblasts still remains largely unknown. Consequently, this study aimed to investigate the possible involvement of resistin, visfatin and ghrelin in molecular alterations in cementoblasts exposed to chemically induced hypoxia.

## 2 MATERIALS AND METHODS

### 2.1 Cell culture

Immortalized mouse cementoblast cells (OCCM‐30)^19^, ^20^ were kindly provided by Prof. Martha J. Somerman (NIH, NIDCR, Bethesda, Maryland). Cells were grown in α‐MEM (11095–080, Gibco) supplemented with 10% foetal bovine serum (FBS) (10270–106, Gibco) and 1% penicillin/streptomycin (15140–122, Gibco) and incubated in a humidified atmosphere of 5% CO_2_ at 37℃ as previously described.^20^ The cells were seeded into 6‐well plates (657160, Greiner Bio‐one) in a density of 3 × 10^4^ cells/well and the medium was changed twice a week.

### 2.2 In‐vitro hypoxic condition induction

The cobalt (II) chloride hexahydrate (CoCl_2_) (Cat. N°: C8661, Sigma‐Aldrich) was dissolved directly to the growth medium and sterilized through a sterile 0.2 μm spare membrane filter (Z333905‐1EA, Merck) to reach final concentrations of 100 μM or 420 μM, which is based on the hypoxic concentration used by He et al.,^21^ respectively. Cementoblasts were cultivated supplemented with 100 μM or 420 μM CoCl_2_ for indicated time periods to mimic the different hypoxic culture conditions.^9^ Cells cultured without CoCl_2_ served as the normoxic control.

### 2.3 Reagents

Cells were stimulated using mouse recombinant resistin (Cat. N°: SRP4560, Sigma‐Aldrich), visfatin (Cat. N°: SRP4908, Sigma‐Aldrich) and ghrelin (Cat. N°: 494127, Sigma‐Aldrich) at a concentration of 100 ng/ml.

The 1.0 μg/ml ERK1/2 inhibitor (FR180204) (#328007, Calbiochem) and 3.67 μg/ml HIF‐1α inhibitor (IDF‐11774) (HY‐111387, MedChemExpress) were used. 1.0 μg/ml DimethyIsulphoxide (DMSO) (D8418‐50ML, Sigma‐Aldrich) was used as negative‐control group.

### 2.4 Small interfering RNA oligonucleotides

The specific siRNAs targeting mouse HIF‐1α (SI00193774) (sense 5’‐ACGAAGCGTTTCACAGCTTAA‐3’), negative control (1027280) and cell death control (SI04939025) along with their corresponding antisense RNA oligonucleotides were purchased from QIAGEN (Germany). Transfection of the 1.2 µl siHIF‐1α oligonucleotides was performed by incubation with 12 µl HiPerFect^@^ Transfection Reagent (301705, QIAGEN) in 100 µl Opti‐MEM medium (31985–062, Gibco) at room temperature (RT) for 10 min. Every transfection mixture was added into 2.3 ml growth medium to reach a final concentration of 125 ng/ml siHIF‐1α in the 6‐well plate in which OCCM‐30 cells were cultured at 60%–70% confluence. After transfection for 24 hours, resistin, visfatin and ghrelin were added in a concentration of 100 ng/ml, respectively.

### 2.5 Isolation of total RNA and reverse transcription (cDNA synthesis)

Total RNA was extracted using the NucleoSpin^@^ RNA Kit (740955.50, MACHEREY‐NAGEL). The quality and quantity of the eluted mRNA were measured for optical density photometrically using a spectrophotometer (Nanodrop2000, Thermo Scientific). cDNA was synthesized from 1.0 μg of total RNA using the iScript^TM^ cDNA Synthesis Kit (#1708891, Bio‐Rad) and performed in the CFX96^TM^ System Cycler (Bio‐Rad).

### 2.6 Quantitative real‐time polymerase chain reaction (RT‐PCR)

For RT‐PCR reaction, the SsoAdvanced^TM^ Universal SYBR^@^ Green Supermix (#1723271, Bio‐Rad) was used. The primers employed were as follows: *HIF*‐*1α* (qMmuCID0005501), *caspase*‐*3* (qMmuCED0047599), *caspase*‐*8* (qMmuCID0005542) and *caspase*‐*9* (qMmuCED0046922) all from Bio‐Rad. All reactions were run in triplicate, and expression was normalized to that of the housekeeping gene *glyceraldehyde*‐*3*‐*phosphate dehydrogenase* (*GAPDH*) (qMmuCED0027497, Bio‐Rad). Results were analysed using the Bio‐Rad CFX Manager 3.1 software. Relative levels of transcript expression were quantified using the 2^ΔΔCt^ method.^22^

### 2.7 Protein extraction and Western blot analysis

RIPA buffer (89901, Thermo Scientific) supplied with 3% protease inhibitor (78442, Thermo Scientific) was used for protein extraction. Protein concentrations were measured using Pierce^TM^ BCA Protein Assay Kit (23225, Thermo Scientific). Further, equal amounts of protein (20 µg/lane) were separated using 10% sodium dodecyl sulphate‐polyacrylamide (SDS‐PAGE) gel by electrophoresis and followed by transferring to a nitrocellulose membrane (1704271, Bio‐Rad).

The membranes were then blocked with 5% non‐fat milk (T145.1, ROTH) solution in 1 × TBS (20 mM Tris, 500 mM NaCl, pH 7.5) with 0.1% Tween‐20 (P1379, Sigma‐Aldrich) for 1 hour at RT and then incubated with the primary antibodies against hydroxy‐HIF‐1‐alpha (Pro564) (#3434, Cell Signalling Technology); HIF‐1‐alpha (#14179, Cell Signalling Technology); caspase‐3 (#9662, Cell Signalling Technology); caspase‐8 (#4790, Cell Signalling Technology); caspase‐9 (#9504, Cell Signalling Technology); ERK1/2 (MBS8241746, BIOZOL) and phospho‐ERK1/2 (44‐680G, Thermo‐Fisher) in a concentration of 1:1000.

The antibody against β‐actin (ab8227, Abcam) was used to standardize the loading. The blots were then employed with the horseradish peroxidase‐conjugated secondary antibodies: Polyclonal goat anti‐rabbit (P0448, Dako); rabbit anti‐goat (P0160, Dako) and polyclonal goat anti‐mouse (P0447, Dako) in a concentration of 1:2000. The band signals were visualized with X‐ray Amersham Hyperfilm (28906836, GE Healthcare) utilizing Amersham ECL Western Blotting Detection Reagents (9838243, GE Healthcare) and visualized using the OPTIMAX X‐Ray Film Processor (11701–9806–3716, PROTEC GmbH). ImageJ software (version 1.62, National Institutes of Health, USA) was used to quantify the signal intensity.

### 2.8 Annexin‐V/propidium iodide apoptosis assay by FACS

Apoptosis induction was verified after treatment with COCl_2_ using an FITC Annexin‐V/Propidium Iodide (PI) Apoptosis Detection Kit (#556547, BD Biosciences, Europe). Briefly, both floating and adherent cells were harvested with Gibco™ StemPro Accutase Cell Dissociation Reagent (A1110501, Gibco). The samples were washed twice with 1×phosphate‐buffered saline (PBS) (10010023, Thermo‐Fisher) and adjusted to a concentration of 1 × 10^6^ cells/ml in pre‐cooled 1 × PBS. Control groups used for compensation and quadrants were set up with unstained, single FITC Annexin‐V staining and single PI staining cells. For staining control and experimental groups, cells were stained with 5 μl FITC Annexin‐V and 5 μl PI staining solution. Following incubation for 15 min at RT in the dark, 400 μl of 1 × binding buffer was added to each tube. Finally, cells were kept on ice and analysed for apoptosis and necrosis using a FACS Vantage Flow Cytometer (SP6800 Spectral Analyzer, Sony Biotechnology, Berlin, Germany) within 1 hr.

The percentages of the different cell populations were processed in the different quadrants in an FITC Annexin‐V/PI dot plot using SP6800 Spectral Analyzer software (version 2.0.2, Sony corporation). For every sample, 1 × 10^4^ events were recorded and this assay was done in triplicate. Cells that were FITC Annexin‐V ^neg^/PI ^neg^ were considered being alive, cells being a FITC Annexin‐V ^pos^/PI ^neg^ were apoptotic and cells being FITC Annexin‐V ^pos^/PI ^pos^ were necrotic.

### 2.9 Statistical analysis

Statistical analyses were performed using GraphPad Prism 8.0 software (GraphPad software Inc., San Diego, CA, USA). All values are expressed as means ±standard deviation (SD) and analysed using student's *t* test for unpaired samples to determine the statistically significant differences between two groups. A one‐way ANOVA was used for multiple comparisons involving more than two groups. Differences were considered statistically significant at a *p*‐value of <0.05. Data distribution was analysed using the Kolmogorov‐Smirnov and the Shapiro‐Wilk test and visually using QQ plots. Each experiment was performed in triplicate and repeated successfully at least three times.

## 3 RESULTS

### 3.1 CoCl_2_‐mimicked hypoxia and its effects on apoptosis and necrosis of cementoblasts

**FIGURE 1:**
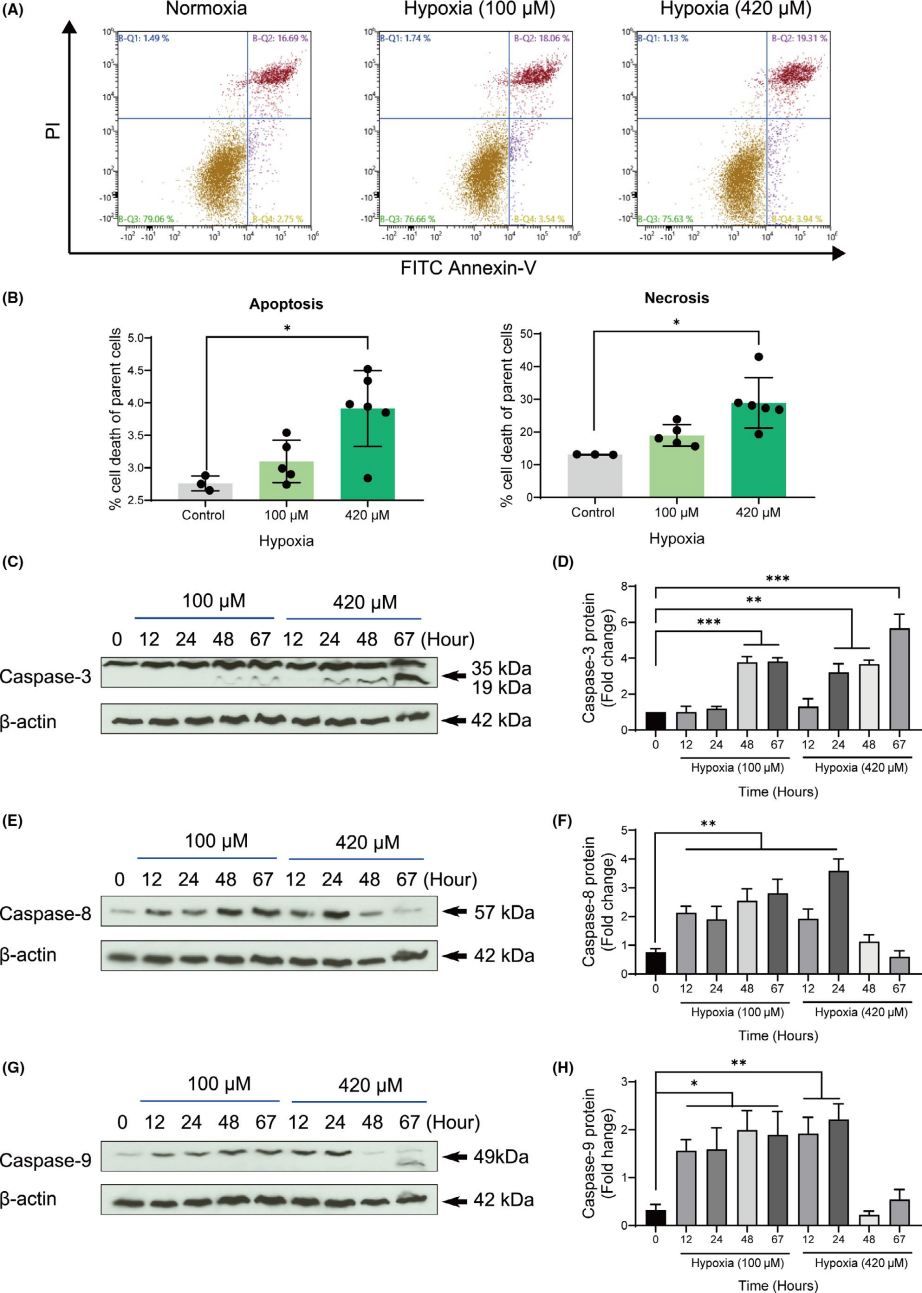
Evaluation of CoCl_2_‐mimicked hypoxia and its effects on apoptosis and necrosis of OCCM‐30 cells. (A) Cell apoptosis and necrosis exposed to cobalt (II) chloride (CoCl_2_)‐induced hypoxia (100 μM or 420 μM) were measured by flow cytometry using the FITC Annexin‐V/PI apoptosis assay. The lower right section of the four different quadrants represents apoptosis (Annexin‐V^pos^/PI^neg^) and the upper right represents necrosis (Annexin‐V^pos^/PI^pos^). (B) The rates of apoptosis and necrosis were measured by flow cytometry. Treatment under 420 μM hypoxic condition increased levels of apoptosis and necrosis. The student *t* test was performed for the comparison and significant differences between the groups are indicated as *p < 0.05, *n* = 3. (C), (E), (G) The OCCM‐30 cells were incubated for various periods in hypoxic environment (100 μM or 420 μM). Total cell lysates were subjected to western blot analysis using specific antibodies for caspase‐3, *caspase*‐8 and *caspase*‐9. Increase in caspase‐3, *caspase*‐3 and *caspase*‐9 in response to hypoxia (100 μM or 420 μM) in OCCM‐30 cells are observed. Results showed that hypoxia increase the expression of caspases in a time‐ and concentration‐dependent manner. (D), (F), (H) Graphs show the densitometric quantification ratio of WB results. The data are expressed as percentage relative to controls (fold change) not exposed to hypoxia. The blots and photomicrographs are representative for three independent experiments. The intensity of signals is expressed as arbitrary units. The data are plotted as the mean ±SD. Significant differences with the control are indicated as follows: Ns =not significant (*p* < 0.05), *p < 0.05, **p < 0.01 and ***p < 0.001

To validate our in‐vitro model of hypoxic conditions, we examined the effect of different concentrations of CoCl_2_‐induced hypoxia (100 or 420 μM) on apoptosis ratio of cementoblasts by flow cytometric analysis. The results obtained using FACS demonstrated that the apoptosis rate was significantly elevated in the 420 μM hypoxic group (3.91% ±0.53%) compared with the control group (2.75% ±0.3%) (*p* < 0.05). Similarly, the necrosis rate was significantly elevated in the 420 μM hypoxic group (28.9% ±7.04%) compared with the control group (18.94% ±3.44%) (*p* < 0.05) (Figure 1A, 1B). This indicated a negative effect of CoCl_2_‐mimicked hypoxia on cementoblast cell survival.

**FIGURE 2:**
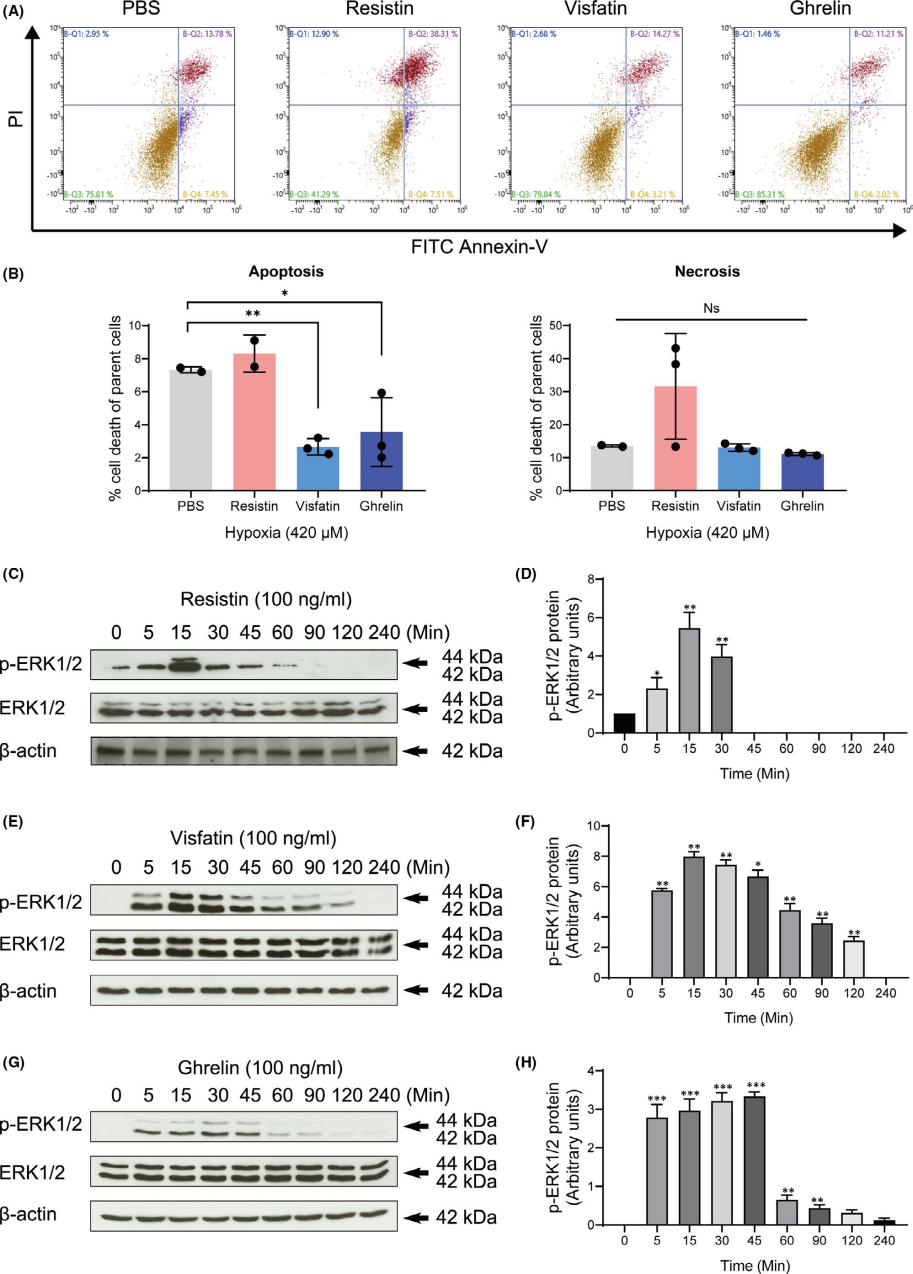
Effects of adipokines on the apoptosis and necrosis of OCCM‐30 cells. (A) The rate of apoptosis and necrosis under expose to different adipokines (resistin, visfatin or ghrelin) were measured by flow cytometry using the FITC Annexin‐V/PI apoptosis assay. For each graph, the lower right section of the four different quadrants represents apoptosis (Annexin‐V^pos^/PI^neg^) and the upper right represents necrosis (Annexin‐V^pos^/PI^pos^). (B) Flow cytometry detection shows that apoptosis was inhibited in response to treatment with visfatin (**p < 0.01) or ghrelin (*p < 0.05). (C), (E), (G) Effect of resistin, visfatin and ghrelin at 100 ng/ml concentration on phosphorylated ERK1/2 expression after indicated periods on cementoblasts cell culture. Western blots showed that resistin‐, visfatin‐ or ghrelin‐induced phosphorylation of ERK1/2 exerting a dual expression on cementoblasts. (D), (F), (H) Graphics represent the relative expression values of p‐ERK1/2 normalized to respective control cells. Data are expressed as percentage relative to controls (fold change) not exposed to hypoxia. The blots and photomicrographs are representative for three independent experiments. The intensity of signals is expressed as arbitrary units. The data are plotted as the mean ±SD. Significant differences with the control are indicated as follows: Ns =not significant (*p* < 0.05), *p < 0.05, **p < 0.01 and ***p < 0.001

The hypoxia concentration at 100 or 420 μM increased had an increasing effect on caspase‐3, *caspase*‐8 and *caspase* −9 expression during 12–24 hr (Figure 2C, 2E, 2G). These results showed that the activated versions of the caspases were markedly increased in response to the CoCl_2_‐mimicked hypoxic conditions in a time‐ and concentration‐dependent manner (Figure 1C–1H), suggesting that caspase‐signalling is partly responsible for the apoptosis under hypoxic conditions in cementoblasts.

### 3.2 Adipokines regulated apoptosis and triggered MAPK protein expression

The regulation of the degree of apoptosis in adipokines after exposure to hypoxia was investigated. Our results showed that visfatin and ghrelin administration significantly reduced the apoptosis rate (2.67% ±0.23%, 3.56% ±1.17%, respectively) compared to the non‐treated group (7.325% ±0.12%) (*p* < 0.01) (Figure 2A, 2B). Resistin induced a slight but not statistically significant elevation of the necrosis rate compared to the non‐treated group (*p* > 0.05) (Figure 2A, 2B).

Resistin induced the phosphorylation of ERK1/2 during 5–45 min in a time‐dependent manner (Figure 2C, 2D). Visfatin promoted the phosphorylation of ERK1/2 in a similar manner. The induction was significantly increased after 5 min of treatment and reached a peak at 15 to 30 minutes (Figure 2E, 2F). Similarly, ghrelin also increased ERK1/2 phosphorylation during 5–45 min (Figure 2G, 2H). These results suggest that three adipokines may mediate the occurrence of hypoxia‐induced apoptosis by activation of ERK1/2.

### 3.3 Effect of ERK1/2 inhibitor on hypoxia‐induced apoptosis and necrosis

**FIGURE 3:**
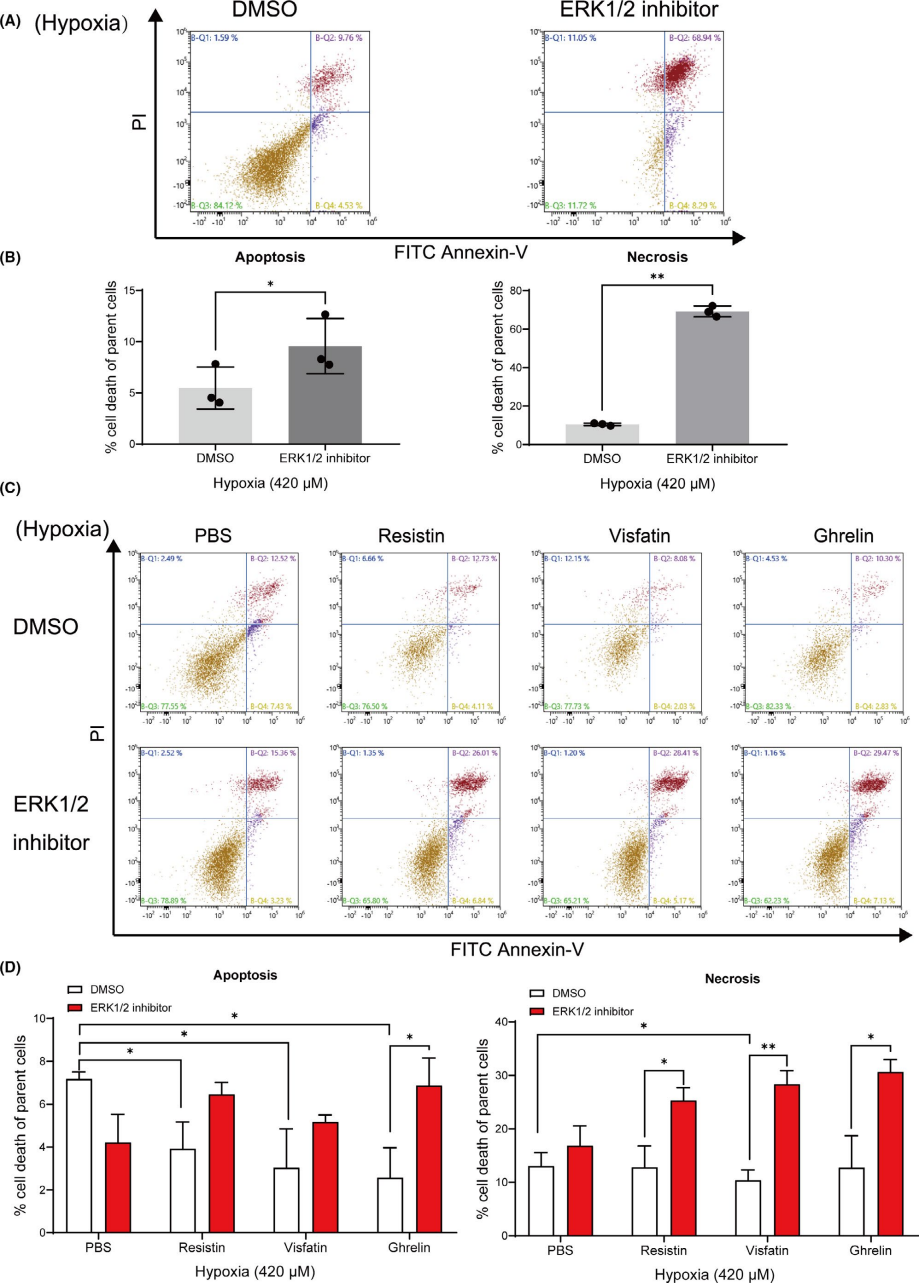
Effect of ERK1/2 blockade on apoptosis and necrosis of cementoblasts exposed to CoCl_2_‐induced hypoxia. (A), (C) Representative plots from FITC Annexin‐V flow cytometry and PI staining experiments performed in triplicate are shown. Apoptotic cells (Annexin‐V ^pos^/PI ^neg^) are shown in the lower right quadrant, and necrosis cells (Annexin‐V ^pos^/PI ^pos^) are shown in the upper right quadrant. (B) Graphics show the percentages of apoptotic and necrotic cells exposed to 1.0 μg/ml ERK1/2 inhibitor (FR180204). Results show that ERK1/2 inhibition significantly increased the apoptosis (*p < 0.05) and also the necrosis rate in cells that exposed to the ERK1/2 inhibitor (**p < 0.01). (D) Graphs shows that resistin, visfatin or ghrelin downregulated the apoptosis percentage of cells. These effects were reversed after the addition of an ERK1/2 inhibitor (*p < 0.05). The necrosis rate was enhanced by the ERK1/2 inhibitor despite the presence of resistin, visfatin or ghrelin (*p < 0.05). The data and photomicrographs are representative for three independent experiments. The data are plotted as the mean ±SD. Significant differences with the control are indicated as follows: Ns =not significant (*p* < 0.05), *p < 0.05, **p < 0.01 and ***p < 0.001

To explore whether the apoptotic response to hypoxia in cementoblasts was mediated by adipokines and specifically by ERK1/2, cells were cultured under hypoxia with or without ERK1/2 inhibitor. As shown in Figure 3A, 3B, on selective suppression of ERK1/2, a significant increase in the degree of hypoxia‐induced apoptosis (9.57% ±2.20% vs. 5.47% ±1.68%), as well as necrosis (69.17% ±2.30% vs. 10.48% ±0.53%), was demonstrated in cementoblasts (Figure 3A, 3B).

Under 420 μM hypoxic condition, the rate of apoptosis in cells cultured in the presence of resistin, visfatin or ghrelin was nearly 50% less compared to the control. This effect was significantly reversed by the addition of an ERK1/2 inhibitor (Figure 3C, 3D). Especially, visfatin significantly inhibited cell apoptosis compared to control cells, but this effect was also highly reversed by the ERK1/2 inhibitor (Figure 3C, 3D). These findings confirmed that an increased apoptosis ratio was due to ERK1/2 blockade under hypoxia. The investigated adipokines have a varying level of protective effect regarding apoptosis initiation and progress.

### 3.4 ERK1/2 is involved in hypoxia‐induced modulation of HIF‐1α expression and its effect are modulated by adipokines

**FIGURE 4:**
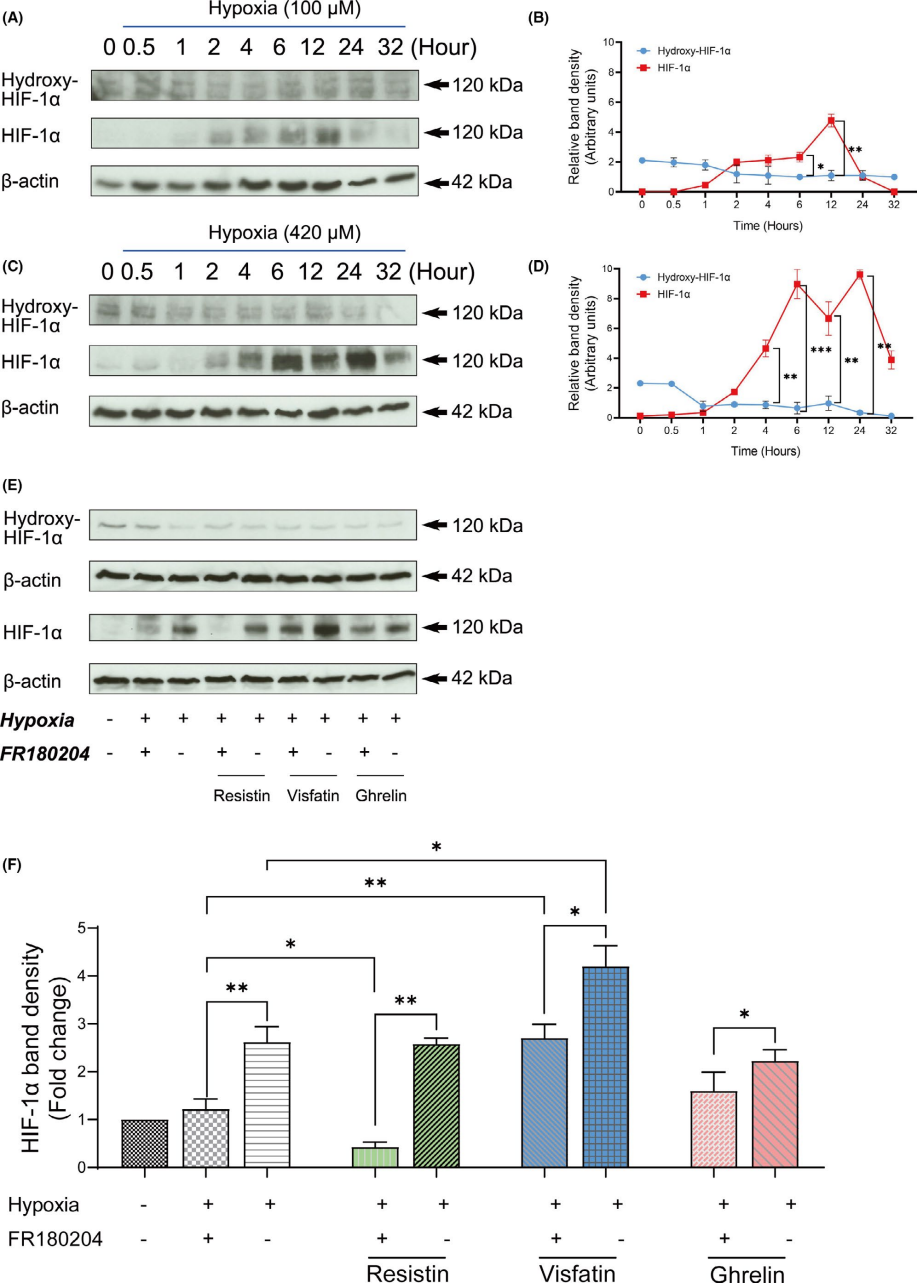
Effect of hypoxia as well as ERK1/2 inhibitor on HIF‐1α expression in cementoblasts. OCCM‐30 cells were cultured under CoCl_2_‐induced hypoxia (100 μM or 420 μM) for the indicated time periods. (A), (C) Western blot showed that the protein expression of HIF‐1α was upregulated by hypoxic conditions in parallel with downregulation of the hydroxy‐HIF‐1α in a time‐dependent manner. β‐actin was loaded as an internal control. (B), (D) Densitometric analysis of band intensities (arbitrary units) of every protein shown as line chart, indicating the relative relation between hydroxy‐HIF‐1α and HIF‐1α expression. (E) OCCM‐30 cells were pretreated with the 1.0 μg/ml ERK1/2 inhibitor (FR180204) for 1 hr, thereafter 100 ng/ml of resistin, visfatin or ghrelin was added. The ERK1/2 pharmacological blockade caused a promotive effect on the expression of hypoxia‐induced HIF‐1α protein (**p < 0.01). Different effects were observed after addition of the adipokines: Resistin decreased the HIF‐1α expression (**p < 0.01), ghrelin induced a moderate promotive effect on the hypoxia‐induced HIF‐1α expression (*p < 0.05), whereas visfatin exhibited a strongly increasing effect on its expression (*p < 0.05). However, these effects were reverted after addition of an ERK1/2 inhibitor (*p < 0.05). (F) Graphics show the HIF‐1α band density (fold change) when cells were exposed to ERK1/2 inhibitor in the presence or absence of adipokines (resistin, visfatin or ghrelin). Data represent results of three independent experiments. The intensity of signals was expressed as arbitrary units. The data are plotted as the means ±SD. Significant differences are indicated as follows: Ns =not significant (*p* < 0.05), *p < 0.05, **p < 0.01 and ***p < 0.001

In a next step, the apoptotic regulation activities of three adipokines were investigated by evaluating the relation between ERK1/2 and HIF‐1α protein levels involved in the hypoxia‐induced apoptosis process. Western blots analysis showed that the expression of HIF‐1α hydroxylation is suppressed by hypoxia after 1‐hr incubation, which is a signal for the activation of HIF‐1α (Figure 4A, 4C). Accordingly, the HIF‐1α protein expression levels were upregulated during 4–12 hr in response to 100 μM hypoxia (Figure 4A, 4B). Moreover, 420 μM hypoxia promotes its expression during 4–24 hr (Figure 4C, 4D).

Co‐treatment with three investigated adipokines revealed that resistin, visfatin and ghrelin promote the HIF‐1α expression under hypoxia conditions (Figure 4E, 4F). The ERK1/2 inhibitor decreased the expression of HIF‐1α despite the addition of resistin, visfatin and ghrelin (Figure 4E, 4F). These data demonstrate the positive relationship between ERK1/2 and HIF‐1α signalling, showing that under hypoxic condition adipokines upregulate the HIF‐1α expression partially via ERK1/2.

### 3.5 Effect of HIF‐1α inhibitor on hypoxia‐induced necrosis

**FIGURE 5:**
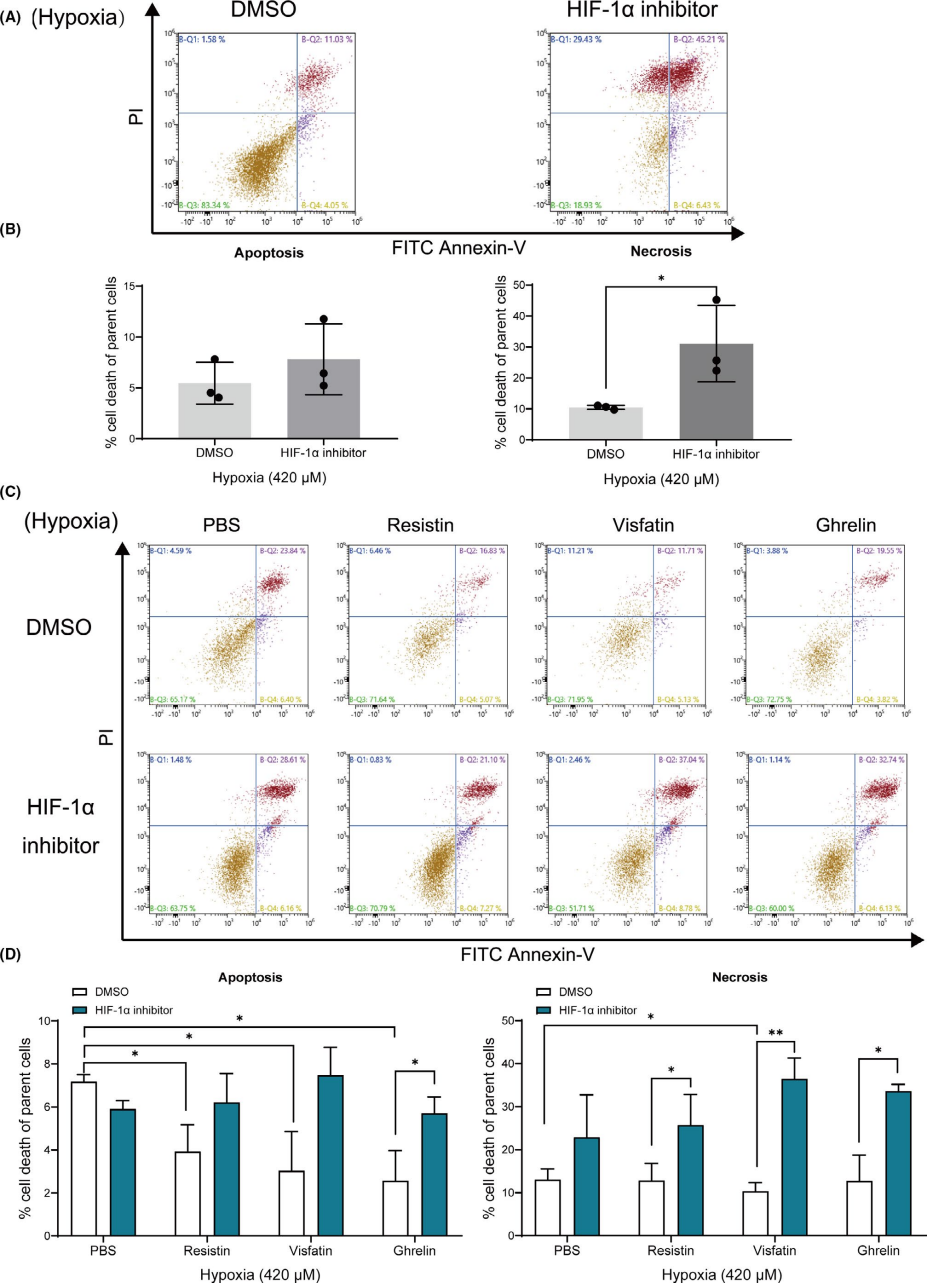
Effect of HIF‐1α inhibition on the hypoxia‐induced necrosis. Cells were preincubated with 3.67 μg/ml HIF‐1α inhibitor (IDF‐11774) for 1 hr and then stimulated with resistin (100 ng/ml), visfatin (100 ng/ml) or ghrelin (100 ng/ml), respectively. Cells were then harvested and stained with FITC Annexin‐V and PI. (A), (C) Percentages of apoptotic and necrotic cells were measured using flow cytometry. Cells being Annexin‐V ^pos^/PI ^neg^ were considered as apoptotic and Annexin‐V ^pos^/PI ^pos^ as necrotic. (B) Graphs show that HIF‐1α inhibitor significantly promotes the necrosis process of OCCM‐30 cells whilst exposing them to CoCl_2_‐induced hypoxia (420 μM) (*p < 0.05). (D) Graphs show that resistin, visfatin or ghrelin downregulated the apoptosis process of cells (*p < 0.05) under 420 μM hypoxic condition. These effects were reversed after HIF‐1α inhibitor supplementation in different concentrations (*p < 0.05). The necrosis was enhanced by HIF‐1α inhibition despite the presence of resistin, visfatin or ghrelin (*p < 0.05). The data and photomicrographs are representative for three independent experiments. The data are plotted as the mean ±SD. Significant differences with the control are indicated as follows: Ns =not significant (*p* < 0.05), *p < 0.05, **p < 0.01 and ***p < 0.001

We found HIF‐1α inhibitor significantly increased the necrosis ratio of cementoblast cultured under 420 μM hypoxic condition (Figure 5A, 5B). Also, under 420 μM hypoxia all three adipokines inhibited the cells apoptotic rate, whilst only visfatin showed an inhibitory effect on the necrosis progress of cementoblasts (Figure 5C, 5D). These inhibitory effects were significantly reversed by an HIF‐1α inhibitor (Figure 5C, 5D). Thus, the present results show that HIF‐1α protein mediates the hypoxia‐induced apoptosis and necrosis process, indicating that adipokines are involved in this regulation via HIF‐1α signalling in cementoblasts.

### 3.6 Effect of HIF‐1α on the expression of apoptosis signalling

**FIGURE 6:**
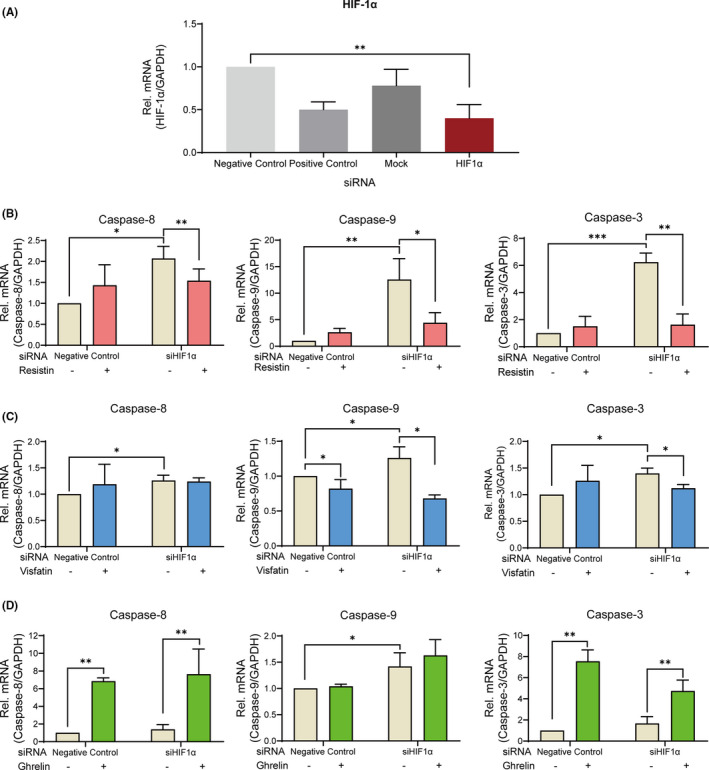
Involvement of HIF‐1α signalling in the apoptotic regulation of cementoblasts under CoCl_2_‐induced hypoxia. (A) The efficacy of siRNA transfections targeting *HIF*‐*1α* was analysed by qRT‐PCR. (B) Single knocking down of *HIF*‐*1α* under 420 μM hypoxia showed an increasing effect on caspase‐8 (*p < 0.05), caspase‐9 (**p < 0.01) and caspase‐3 (***p < 0.001) gene expression, but resistin significantly decreased these gene expressions (**p < 0.01). (C) Silencing of HIF‐1α slightly upregulated caspase‐8, caspase‐9 and caspase‐3 gene expression (*p < 0.05). Visfatin decreased the caspase‐9 gene expression (*p < 0.05). The administration of HIF‐1α siRNA caused further decrease on the gene expression of caspase‐9 (*p < 0.05). (D) Silencing of HIF‐1α caused an increased gene expression of caspase‐9 (*p < 0.05). After addition of ghrelin, caspase‐8 and caspase‐3 gene expression was significantly upregulated (**p < 0.01). The data and blot are representative for three independent experiments. The data are plotted as the mean ±SD. Significant differences with the control are indicated as follows: Ns =not significant (*p* < 0.05), *p < 0.05, **p < 0.01 and ***p < 0.001

Suppression of the *HIF*‐*1α* gene expression using small interfering RNA showed a significant decrease by 59 ± 22% compared to negative‐control group (Figure 6A). Silencing of *HIF*‐*1α* upregulated caspase‐8, caspase‐9 and caspase‐3 gene expression under 420 μM hypoxia (Figure 6B–6D). Furthermore, resistin significantly downregulated these gene expressions (Figure 6B). Visfatin decreased the gene expression of caspase‐9 (Figure 6B). On the contrary, ghrelin increased the gene expression of caspase‐8 and caspase‐3 (Figure 6C). In the presence of ghrelin, silencing of HIF‐1α induced an increasing expression of caspase‐9 (Figure 6D). In toto, CoCl_2_‐mimicked hypoxia requires HIF‐1α to maintain cell survival and adipokines regulate the apoptosis process via crosstalk of ERK1/2 and HIF‐1α.

## 4 DISCUSSION

In this study, we showed that the CoCl_2_‐mimicked hypoxia promotes the HIF‐1α expression and activates the apoptosis pathway. HIF‐1α plays a critical role as a key factor in the survival of cementoblasts. ERK1/2 induced by adipokines reinforces the HIF‐1α expression to further cause an inhibition effect on apoptosis by decreasing caspase‐signalling expression. Thus, HIF‐1α allows cementoblasts to resist apoptosis and plays a vital role in the interaction of ERK1/2 and caspase signalling under lengthy hypoxia when exposed to these three adipokines.

Hypoxia may induce apoptosis through activation of caspases,^23^ which controls initiation and execution of apoptosis.^24^ The caspase activation is triggered by two interrelated pathways: caspase‐8 is a key player for the extrinsic pathway^25^ and caspase‐9 activation is of importance in the intrinsic apoptosis pathway.^26^ Both caspase‐8 and caspase‐9 activate downstream effector caspases including caspase‐3 and caspase‐7 to execute the final steps of apoptosis.^27^ In our study, we observed that the protein of caspase‐3, caspase‐8 and caspase‐9 were highly upregulated under hypoxic conditions, indicating that both apoptosis pathways were activated by hypoxia in cementoblasts.

CoCl_2_ is widely used as a hypoxia mimetic and is known to induce the change in the transcriptional of some genes, such as hypoxia‐inducible factor‐1α (HIF‐1α), p53 and p21.^28^ Recent evidence suggested that HIF‐1α does not mediate all the effects of hypoxia, but it is an important part of this chemical cellular response.^29^ The use of CoCl_2_ allows us to distinguish the hypoxia effect that occurs specifically through HIF‐1α. Therefore, CoCl_2_‐induced apoptosis may be a simple and convenient in‐vitro model for investigating the molecular mechanisms in hypoxia‐linked cell apoptosis. Our study aimed to investigate whether the HIF‐1α and ERK1/2 activation was involved in CoCl_2_‐induced apoptosis in OCCM‐30 cells and to expound on the underlying mechanisms. Therefore, we use this chemical‐induced hypoxia as stimulation source instead of physiological hypoxia.

Several investigators have identified HIF‐1α as a critical apoptotic mediator expressed mainly under a hypoxic condition,^30^ which allows cells to survive through a variety of cellular biological regulations.^31^, ^32^ Sasabe et al.^33^ showed that HIF‐1α prevented apoptotic cell death through an inhibition of cytochrome C release and the activation of Akt and ERK1/2. HIF‐1α is rapidly degraded under normoxic conditions,^34^ whereas it prevents hydroxylation and steadily expresses HIF‐1α under hypoxic conditions.^35^ Thus, its activity increases in hypoxia stimulated cells.^31^ The hydroxy‐HIF‐1α itself is the limiting factor for HIF‐1α degradation.^36^ In the present study, we found that the OCCM‐30 cell line expressed an increased level of HIF‐1α and a decreased level of hydroxy‐HIF‐1α under a hypoxic condition in a time‐dependent manner.

Zhu et al.^37^ reported that hypoxia induced by 100 μM CoCl_2_ resulted in stable HIF‐1α protein expression in immortalized osteocyte‐like cells in vitro. In this study, we also found that apoptosis was markedly increased by an HIF‐1α inhibitor (IDF‐11774), suggesting that HIF‐1α is one of the key mediators for cell survival of cementoblasts. Hence, cementoblasts are self‐resistant to hypoxia by inducing HIF‐1α expression.

To elucidate the relevant molecules that participate in hypoxia‐induced apoptosis, we then aimed to investigate whether the activation of ERK1/2 by these three adipokines is involved in the hypoxia‐induced HIF‐1α expression. Indeed, the present study revealed that pre‐conditioning with an ERK1/2 inhibitor (FR180204) impairs the HIF‐1α stabilization in hypoxia indicating its involvement in the effects caused by the adipokines. Thus, ERK1/2 is essential and associated with the modulation of HIF‐1α during hypoxia. These findings are consistent with Mottet et al. (2002) who showed that ERK1/2 activation enhanced HIF‐1α activity in the hypoxia signal transduction pathway.^38^

We also demonstrated that IDF‐11774 was able to promote the gene expression of caspases under hypoxia. Moreover, it was shown that HIF‐1α silencing promotes *caspase*‐*3*, c*aspase*‐*8* and c*aspase*‐*9* gene expression, indicating the anti‐apoptotic effect of HIF‐1α in hypoxic conditions on cementoblasts. These results suggest that HIF‐1α is involved in both the extrinsic and the intrinsic apoptosis pathways to maintain cementoblast haemostasis.

**FIGURE 7:**
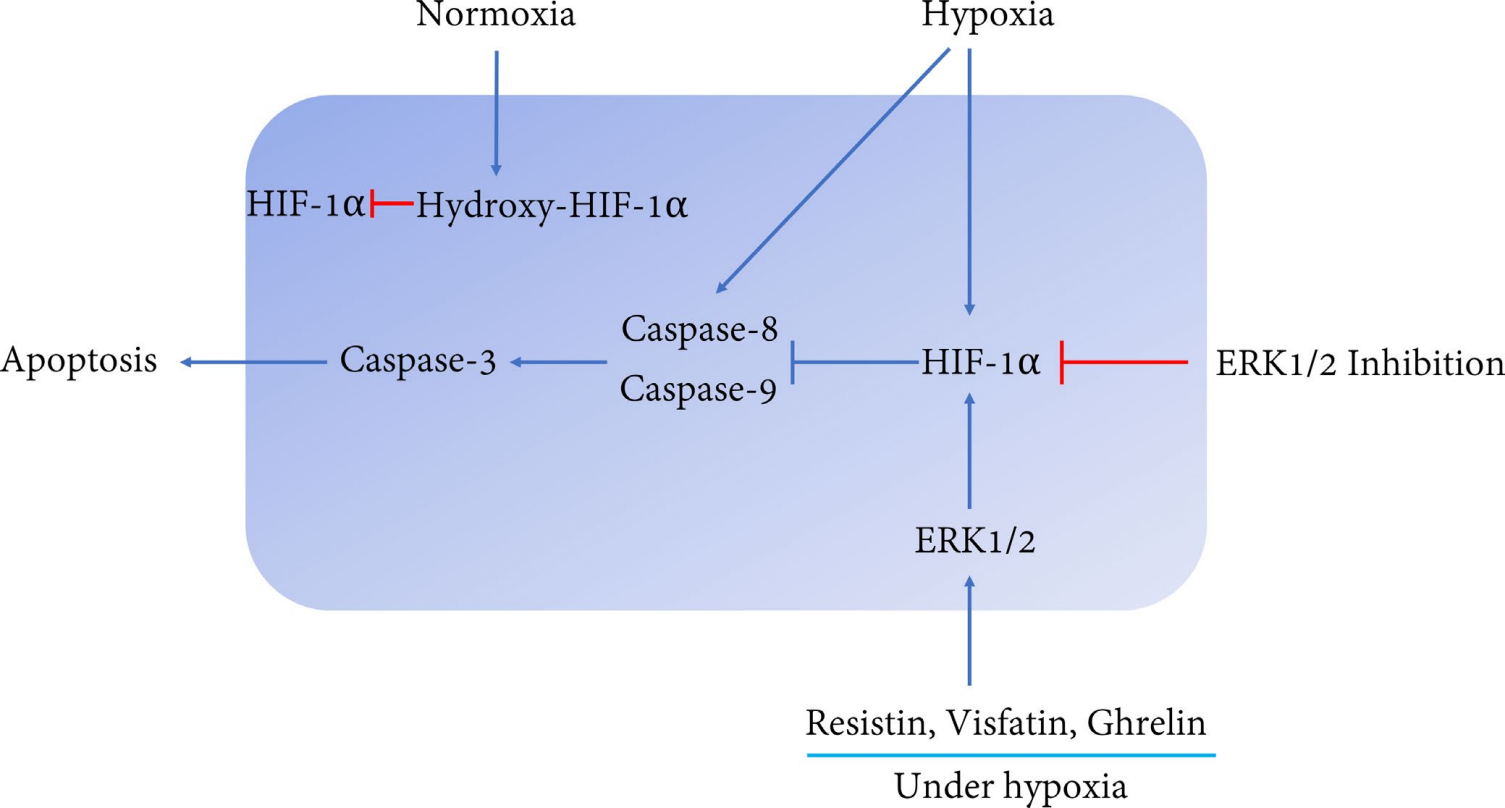
Proposed molecular interactions between ERK1/2 and HIF‐1α signalling to apoptotic pathways on OCCM‐30 cementoblasts cultivated under chemical‐induced hypoxic condition

With regard to these pathways, Allan et al.^39^ showed that ERK1/2 suppresses apoptosis by inhibition of caspase‐9 and subsequent caspase‐3. Hartel et al.^40^ found that MEK/ERK1/2‐mediated inhibition of the caspase‐3 protects endothelial cells against apoptosis under transient hypoxia. Here, FR180204 enhanced the apoptosis of cementoblasts, indicating that ERK1/2 participates in the apoptosis regulation in cementoblasts. In addition, we observed that HIF‐1α is stable in the presence of three adipokines. We conclude that CoCl_2_‐mimicked hypoxia requires HIF‐1α participation to maintain cell survival. Furthermore, our data demonstrated that resistin, visfatin and ghrelin activate ERK1/2, thus, regulating the apoptosis process through a crosstalk between ERK1/2 and HIF‐1α signalling (Figure 7). This is consistent with a previous study, which showed that ERK1 directly participates in HIF‐1α activation response to hypoxia in endothelial cells.^41^ Therefore, ERK1/2 can be considered the key elements involved in the regulation of the apoptosis pathway and HIF‐1α is mandatory to inhibit hypoxia‐induced apoptosis.

However, it should be noted that murine cementoblasts are probably not fully comparable to human primary cementoblasts so that investigations on primary human material are mandatory before patient studies.

The apoptosis of cementoblasts is known to be of critical importance during OTM. We propose the hypothesis that in cementoblasts the imbalance of the three tested adipokines induced an impairment of ERK1/2 activity, the alteration of HIF‐1α pathways would unleash the miss‐regulated cementoblast apoptosis. Alternatively, if reagents were able to regulate the balance of adipokines surrounding the cementoblasts, the changes in the regulation of ERK1/2 and HIF‐1α might not be lethal for cells.

## 5 CONCLUSION

In conclusion, the HIF‐1α is a key mediator involved in the inactivation of the apoptosis pathway, whilst resistin, visfatin or ghrelin active ERK1/2 to regulate HIF‐1α expression. Under hypoxic conditions, these three adipokines contribute to cell survival partially through this crosstalk mechanism. Our study, therefore, establishes a link between the impact of these three adipokines (resistin, visfatin and ghrelin) and hypoxia‐induced apoptosis processes under hypoxia during OTM. This might provide new insight into difficulties in the orthodontic treatment of obese patients in the long term.

## CONFLICT OF INTEREST

The authors have stated explicitly that there are no conflicts of interest in connection with this article.

## AUTHOR CONTRIBUTIONS

**Jiawen Yong:** Conceptualization (equal); Data curation (equal); Formal analysis (equal); Methodology (equal); Software (equal); Validation (equal); Writing‐original draft (equal). **Julia von Bremen:** Formal analysis (equal); Project administration (equal); Supervision (equal); Writing‐review & editing (equal). **Sabine Groeger:** Investigation (equal); Methodology (equal); Project administration (equal); Validation (equal); Writing‐review & editing (equal). **Gisela Ruiz‐Heiland:** Methodology (equal). **Sabine Ruf:** Formal analysis (equal); Funding acquisition (equal); Methodology (equal); Project administration (equal); Supervision (equal); Validation (equal); Writing‐review & editing (equal).

## Data Availability

The data underlying this article will be shared upon reasonable request to the corresponding author.
